# Glial Progenitor-Like Phenotype in Low-Grade Glioma and Enhanced CD133-Expression and Neuronal Lineage Differentiation Potential in High-Grade Glioma

**DOI:** 10.1371/journal.pone.0001936

**Published:** 2008-04-09

**Authors:** Johan Rebetz, Dongping Tian, Annette Persson, Bengt Widegren, Leif G. Salford, Elisabet Englund, David Gisselsson, Xiaolong Fan

**Affiliations:** 1 The Rausing Laboratory, Division of Neurosurgery, Lund University Hospital, Lund, Sweden; 2 Strategic Research Center for Stem Cell Biology and Cell Therapy, Lund University, Lund, Sweden; 3 Department of Cell and Molecular Biology, Lund University, Lund, Sweden; 4 Department of Clinical Genetics, Lund University Hospital, Lund, Sweden; 5 Department of Pathology, Lund University Hospital, Lund, Sweden; City of Hope Medical Center & Beckman Research Institute, United States of America

## Abstract

**Background:**

While neurosphere- as well as xenograft tumor-initiating cells have been identified in gliomas, the resemblance between glioma cells and neural stem/progenitor cells as well as the prognostic value of stem/progenitor cell marker expression in glioma are poorly clarified.

**Methodology/Principal Findings:**

Viable glioma cells were characterized for surface marker expression along the glial genesis hierarchy. Six low-grade and 17 high-grade glioma specimens were flow-cytometrically analyzed for markers characteristics of stem cells (CD133); glial progenitors (PDGFRα, A2B5, O4, and CD44); and late oligodendrocyte progenitors (O1). In parallel, the expression of glial fibrillary acidic protein (GFAP), synaptophysin and neuron-specific enolase (NSE) was immunohistochemically analyzed in fixed tissue specimens. Irrespective of the grade and morphological diagnosis of gliomas, glioma cells concomitantly expressed PDGFRα, A2B5, O4, CD44 and GFAP. In contrast, O1 was weakly expressed in all low-grade and the majority of high-grade glioma specimens analyzed. Co-expression of neuronal markers was observed in all high-grade, but not low-grade, glioma specimens analyzed. The rare CD133 expressing cells in low-grade glioma specimens typically co-expressed vessel endothelial marker CD31. In contrast, distinct CD133 expression profiles in up to 90% of CD45-negative glioma cells were observed in 12 of the 17 high-grade glioma specimens and the majority of these CD133 expressing cells were CD31 negative. The CD133 expression correlates inversely with length of patient survival. Surprisingly, cytogenetic analysis showed that gliomas contained normal and abnormal cell karyotypes with hitherto indistinguishable phenotype.

**Conclusions/Significance:**

This study constitutes an important step towards clarification of lineage commitment and differentiation blockage of glioma cells. Our data suggest that glioma cells may resemble expansion of glial lineage progenitor cells with compromised differentiation capacity downstream of A2B5 and O4 expression. The concurrent expression of neuronal markers demonstrates that high-grade glioma cells are endowed with multi-lineage differentiation potential *in vivo*. Importantly, enhanced CD133 expression marks a poor prognosis in gliomas.

## Introduction

Gliomas, the most common primary tumors in the adult central nervous system, are currently classified according to their morphological features, into low- and high-grade glioma. Cells of low-grade (I and II) gliomas are well differentiated with clear histological similarity to astrocyte or oligodendrocyte lineage. High-grade (III and IV) gliomas are more anaplastic, with features resembling immature astrocytes, oligodendrocytes or a mixture of both types. Low-grade gliomas are frequently diagnosed in relatively young patients and many of these eventually develop into anaplastic gliomas which subsequently progress to the so-called secondary glioblastoma (GBM). In contrast, the GBM in older patients are mostly diagnosed as *de novo* without any clinically detectable history. It is unclear whether the early stages of *de novo* GBM development resemble low-grade gliomas. Although previous studies suggested glioma expression of oligodendrocyte progenitor cell antigen NG2 and PDGFRα, and transcription factor Olig1/2 [1]–[3], the lineage commitment and the stage of differentiation blockage of glioma cells are not clarified [4].

For decades, the median survival of high-grade gliomas has not been significantly improved [4]. In efforts to identify crucial cellular and molecular targets for glioma treatment, recent studies have indicated that all grades of gliomas contain putative tumor stem cells, which can be CD133^+^ or CD133^−^ [5], [6]. These cells are endowed with self-renewal and multi-lineage differentiation capacity in neurosphere-forming assay, a surrogate assay for neural stem cells. Particularly, CD133^+^ putative GBM stem cells were capable of xenograft GBM initiation where the CD133^−^ GBM cells from the same patients failed to do so [7]–[9]. Compared with normal neural stem/progenitor cells, glioma derived cells showed an enhanced and more aggressive self-renewal capacity [9]. Thus, neurosphere-forming glioma cells, which may or may not be CD133^+^, are likely to be the crucial targets for successful treatment. However, neurosphere-forming capacity is an *in vitro* growth factor dependent feature common to neural stem cells as well as progenitor cells [10]; it is also likely that cells normally not endowed with such capacity can gain neurosphere-forming capacity due to transformation mechanisms. In fact, glial lineage restricted progenitor cells can *in vitro* be reprogrammed to acquire multi-lineage differentiation capacity in an environmental cue dependent manner [11]. It has been unclear whether glioma cells *in vivo* are endowed with a multi-lineage differentiation potential. Similarly, xeno-transplantation may only assess those tumor cells capable of adapting to growth in a mouse environment [12], [13]. It is therefore controversial whether neurosphere- or xenograft tumor- initiating glioma cells represent the authentic glioma stem cells, from which gliomas originate [14].

In analogy with the well characterized hematopoiesis hierarchy, mature neural cells are derived from the neural stem cells via multiple progressively committed/differentiated intermediate progenitor cells [15]. The differentiation stages along the hematopoietic hierarchy of primitive hematopoietic cells and leukemic cells can, to a significant extent, be inferred from surface marker expression in combination with functional analysis [16]. Similar strategies have been applied to identify the primitive neural cells. For example, cells with CD133^+^CD34^−^CD45^−^CD24^−^ phenotype represent the neural stem/progenitor cells from embryonic human brain tissues [17]. Neuronal lineage restricted precursors can be isolated via E-NCAM expression [18], [19]. Along the glial lineage, the neural stem cell derived immediate progeny are likely the A2B5 expressing glial-restricted precursor (GRP), which are capable of generating oligodendrocytes, type-2 astrocytes and type-1 astrocytes [20], [21]. Other types of glial lineage progenitor cells, likely downstream to GRP, have also been identified via A2B5 expression [20]–[27]. Although the exact *in vivo* relationship between these A2B5 expressing glial precursors has been challenging to establish, *in vitro* studies have demonstrated that GRP can generate oligodendrocyte/type-2 astrocyte progenitor (O-2A) cells and type-1 astrocyte-restricted progenitor (ARP) [21], [28]. The O-2A progenitor cells, which are characterized by cell surface expression of platelet-derived growth factor receptor-α (PDGFRα), A2B5 and O4, can account for 4% of the adult human white matter cells [22], [25], [26]. Via early and late oligodendrocyte progenitors, which gradually lose PDGFRα, A2B5 and O4 expression and gain O1 expression in pre-myelinating oligodendrocytes, O-2A progenitor cells give rise to oligodendrocytes [21], [27], [29]. But O-2A progenitor cells can *in vitro* also give rise to type-2 astrocytes in a growth factor cue dependent manner [26]. Depending on the utility of growth factor combinations, O-2A progenitor cells can also be reprogrammed to acquire neurosphere-forming and neuronal lineage differentiation capacities [11]. The ARPs also express A2B5, but not PDGFRα and O4 [22]–[24], [29]. Human ARPs were also demonstrated to express CD44 [28], [30].

As demonstrated in previous studies, although gene expression of cancer cells reflects genetic and epigenetic alterations, a considerable fraction of this gene expression can nevertheless be characteristic of the non-transformed cell-of-origin. This principle has been applied to distinguish between leukemias originating from either stem cells or from progenitor cells by characterization of cancer initiating capacity and cancer-characteristic genetic mutations in these cell fractions [31], [32]. In this study, we characterized freshly isolated low-grade and high-grade glioma cells for expression of surface markers characteristic of the glial lineage differentiation hierarchy and analyzed the expression of these markers in relation to patient survival. We also studied the glioma cell multi-lineage differentiation potential *in vivo* by assessing GFAP and NSE/synaptophysin expression in the corresponding formaldehyde fixed specimens.

## Materials and Methods

### Tumor specimens, cell processing and flow cytometric analysis

Glioma biopsies of fresh tumor tissue were obtained from patients operated at the Clinic of Neurosurgery, Lund University Hospital, Sweden. Formaldehyde-fixed, paraffin embedded tissue blocks derived from the same surgical excision biopsies were obtained from the Department of Pathology, Lund University Hospital. Patient survival data were obtained from the Swedish National Register of Population (National Board of Health and Welfare). Permission for using these materials was obtained from The Regional Ethical Review Board in Lund, and written informed consent was obtained from patients.

For preparing viable glioma cells, freshly obtained specimen were cut finely into small pieces, treated in IMDM with 0.5 mg/ml collagenase (Sigma) and 25 µg/ml DNAse (Sigma) at 37°C for 40 minutes. Red cells were lysed with NH_4_Cl; the resulting cells were washed in PBS containing 2% FCS. For flow-cytometry analysis, these cells were either used directly or resuspended in freezing medium containing 10% DMSO and 90% FCS for storage in liquid nitrogen. About 5 to 10×10^6^ of the freshly isolated or thawed cells were first incubated with non-specific blocking mouse IgG1 at 50 µg/ml (clone MOPC 21, Sigma) at 4°C for 20 minutes. Subsequently, about 5×10^5^ cells were stained with allophycocyanin (APC)-conjugated anti-CD45 (clone 2D1), anti-CD31 (clone AC114.5) monoclonal antibodies (mAb) in combination with one of the fluorescein isothiocyanate or phycoerythrin (PE)-conjugated mAbs against PDGFRα (clone αR1), CD44 (clone G44-26), CD24 (clone ML5), EGFR (clone EGFR1) or CD133 (clone AC133), or the isotype-matched control mAbs, at saturating concentrations at 4°C for 15 minutes. Subsequently, cells were washed once with PBS and resuspended in 500 µl PBS supplemented with 2% FCS and 1.0 µg/ml 7-aminoactinomycin D (7-AAD, Sigma). The A2B5, O4, and O1 staining were detected using PE-conjugated rat-anti-mouse-IgM (clone Rat(LOU)IgG_2a_) and a staining omitting the unconjugated primary mAb served as control. The multiparameter flow cytometric analysis was performed in a FACScalibur. At least 50 000 events were counted and cell surface expression was analyzed in 7-AAD negatively stained living cells using the Cellquest or Flowjo program. PE-conjugated anti-CD133 and APC-conjugated anti-CD31 mAbs were purchased from Miltenyi Biotec (Bergisch Gladbach, Germany) and unconjugated anti-A2B5 (MAB312R), O4 (MAB345), and O1 mAb (MAB1327) from Chemicon Europe, Ltd. (Hampshire, UK). All other mAbs were purchased from BD Biosciences Immunocytometry Systems. The flow-cytometry analysis was performed at least twice for most of the specimens when sufficient cells were available. CD133 expression was analyzed with two batches of anti-CD133 mAb with comparable data.

### Karyotype analysis of short-term cultured glioma cells

Freshly isolated or thawed glioma cells were cultured in DMDM/F12 medium supplemented with 2% fetal calf serum, basic fibroblast growth factor (20 ng/ml), platelet derived growth factor-AA (20 ng/ml), sonic hedgehoge (2 ng/ml) and 1×N2.

Chromosome banding analysis was performed between passage 3 to 4 by standard methods.

### Immunohistochemistry analysis

Sections of five-µm thickness were mounted on capillary glass slides (DAKO ChemMate Capillary Gap Microscope Slides, 75 mm, DAKO Sweden AB). All sections were microwave pre-treated in 10 mM citrate buffer pH 6.0 for 15 minutes at 800 W in order to achieve antigen retrieval. An automated immunostainer (TechMateTM 500 Plus, DAKO Sweden AB) was used for the staining procedure using DAKO ChemMate Kit Peroxidase/3-3′diaminobenzidine. Primary antibodies used were GFAP (DAKO, polyclonal, 1∶5000 dilution), NSE (Zymed, clone NSE-1G4, 1∶1500 dilution) and synaptophysin (DAKO, polyclonal, 1∶100 dilution). Staining was semi-quantitatively evaluated for the proportion of positively stained versus total cell numbers and the extent of staining intensity. These two variables generally co-vary between samples. The staining was judged as either markedly positive (++), low-moderately positive (+) or negative (−).

## Results

**Figure 1:**
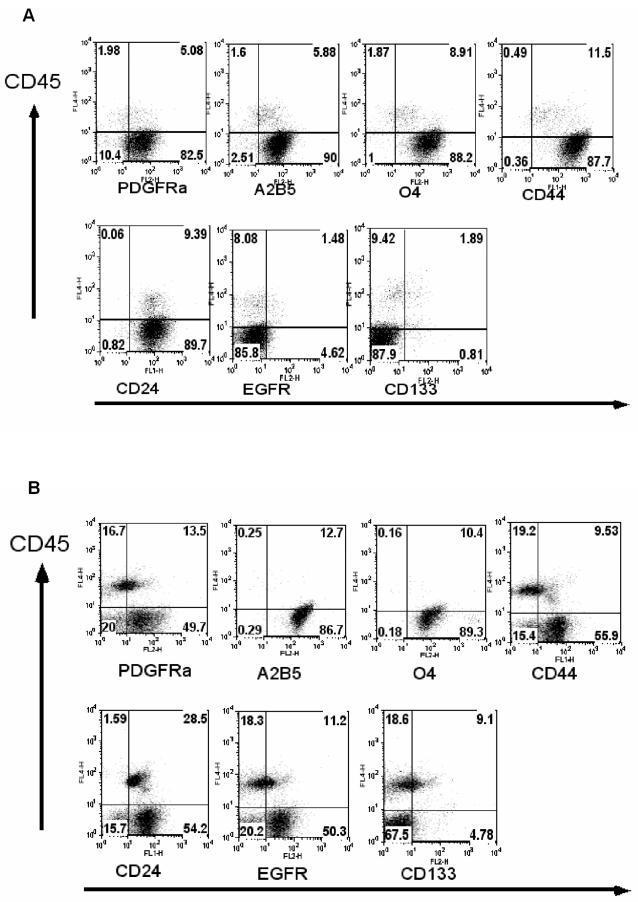
Low-grade glioma cells concurrently express multiple cell surface markers characteristic of adult human glial progenitors. Dot-plot profiles of glial progenitor cell surface markers and CD133 expression on low-grade glioma cells from two representative patients (#1 (A) and #4 (B)) are shown. Freshly isolated glioma cells were simultaneously stained with the indicated antibodies. The hematopoietic cells were distinguished with anti-CD45 staining. The numbers in each quadrate represent the percentages of the cells stained positively or negatively by the respective antibodies.

**Figure 2:**
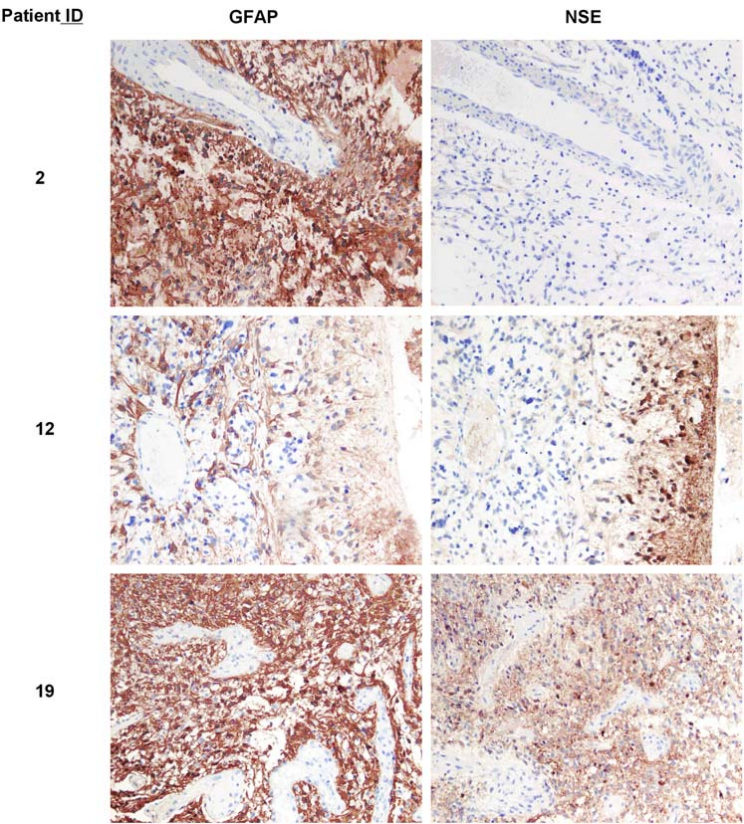
Neuronal marker expression was not detected in low-grade gliomas, but in high-grade gliomas. Staining patterns of GFAP and NSE expression from representative low-glioma (patient ID: #2) and high-grade glioma ((patient ID: #12 and #19) are shown. Original magnification: ×100

We have analyzed the expression of PDGFRα, A2B5, O4 and CD44 as surface markers for glial precursor cells [17], [20]–[24], [27]–[30], and CD133 for stem cells [7], [17] on freshly prepared glioma cells using flow cytometry. In addition, we analyzed the expression of EGFR, which is expressed in immature astrocytes and is critically important for astrocyte development [33]. EGFR is also frequently overexpressed in high-grade glioma cells [34]. Six low-grade and 17 high-grade glioma specimens were analyzed. As shown by representative dot-plot profiles for low-grade glioma specimens (Figure 1), intermingled cells of hematopoietic origin can be distinguished via their CD45 expression. PDGFRα, A2B5, O4, and CD44 were found to be expressed in nearly all CD45 negative cells. The concomitant expression of these progenitor cell markers was observed in nearly all low-grade glioma specimens analyzed (Table 1). These data, combined with the homogenous GFAP staining and the absence of NSE/synaptophysin staining (Table 2 and Figure 2), suggest that low-grade glioma cells are reminiscent of glial-progenitor cells. In 4 out of 6 low-grade glioma specimens, CD133 expression was observed at lower frequencies, but these CD133^+^ cells are predominantly of blood vessel endothelial origin (see below). The expression of EGFR was found in specimens from 2 of the 6 low-grade gliomas. We also analyzed the expression of CD24. Absence of CD24 expression in combination with positive staining on CD133, or PNA or ABCG2/Bcrp1 was used to enrich primitive neural cells with “stem cell-like” properties [17], [35]–[38]. CD24 expression was detected in 4 out of 6 low-grade gliomas.

**Table 1:** Expression of neural stem cell and glial progenitor surface markers in low- and high-grade glioma specimens analyzed.

Patient ID (Age/gender)	Pathological diagnosis	Cells expressing indicated markers (%)							
		PDGFRα	A2B5	O4	O1	CD44	CD24	EGFR	CD133
**Low-grade glioma**									
1 (45/F)	oligoastrocytoma (II)	89	97	99	13	97	99	UD	2.5
2 (6/M)	pilocytic astrocytoma	100	ND	ND	ND	100	UD	UD	UD
3 (7/F)	pilocytic astrocytoma	78	59	96	ND	89	UD	UD	15
4 (36/M)	astrocytoma (II)	71	100	100	11	78	78	71	8
5 (52/M)	astrocytoma (II)	75	79	84	43	76	30	28	8
6 (60/M)	astrocytoma (II)	UD	100	100	29	100	44	UD	UD
**High-grade glioma**									
7 (47/M)	GBM	UD	ND	ND	ND	100	ND	UD	UD
8 (31/M)	anaplastic oligodendroglioma	UD	100	100	18	61	UD	UD	UD
9 (62/M)	GBM	UD	50	UD	ND	100	UD	30	UD
10 (8/M)	anaplastic astrocytoma	32	43	45	32	32	UD	UD	UD
11 (66/M)	GBM	UD	46	17	13	100	UD	UD	UD
12 (36/M)	GBM	45	64	58	ND	26	12	27	10
13 (70/F)	GBM	UD	79	19	ND	96	9	UD	72
14 (47/F)	GBM	63	77	90	ND	92	UD	92	77
15* (38/M)	GBM	96	95	97	66	90	93	80	29
16* (49/M)	oligoastrocytoma III	98	96	96	64	99	98	UD	22
17 (65/M)	GBM	69	100	100	26	85	24	96	90
18 (51/F)	GBM	30	63	74	ND	87	UD	83	16
19 (63/F)	GBM	UD	ND	ND	ND	83	ND	73	70
20 (67/M)	GBM	50	ND	ND	ND	100	UD	100	50
21 (58/F)	GBM	38	74	79	46	88	UD	50	53
22 (61/F)	anaplastic oligodendroglioma	100	100	100	ND	100	100	100	54
23 (55/F)	GBM	38	85	94	83	81	66	72	47
**Other types of brain tumors**									
24 (45/M)	PNET	UD	UD	UD	UD	UD	UD	UD	94
25 (62/M)24 (45/M)	anaplastic ependymoma	UD	25	UD	UD	100	UD	100	15

Freshly prepared or thawed glioma cells were first incubated with non-specific mouse IgG1 mAb, cells were subsequently incubated with APC conjugated anti-CD45 mAb in combination with FITC conjugated anti-CD44, or anti-CD24 mAbs or PE conjugated anti-PDGFRα, anti-EGFR, anti-CD133 mAbs. For A2B5 and O4 staining, cells were incubated with unconjugated A2B5 or O4 mAb respectively and subsequently stained with PE conjugated rat anti-mouse IgM. Cells negatively stained with 7-AAD were analyzed for cell surface marker expression. Data shown are the percentages of CD45 negative cells positively stained for indicated cell surface markers.

* : secondary GBM. UD: undetectable; ND: not done. PNET: primitive neuroectodermal tumor.

**Table 2:** Co-expression of neuronal and glial markers in high-grade glioma cells

Patient ID	GFAP	NSE
1 (45/F)	++	-
2 (6/M)	++	-
3 (7/F)	++	-
4 (36/M)	++	-
5 (52/M)	++	-
6 (60/M)	++	-
7 (47/M)	+++	+
8 (31/M)	++	++
9 (62/M)	+++	++
10 (8/M)	+++	+
11 (66/M)	+++	++
12 (36/M)	+++	+
13 (70/F)	++	++
14 (47/F)	+++	+
15 (38/M)	+++	+
16 (49/M)	+++	+
17 (65/M)	++	++
18 (51/F)	++	+
19 (63/F)	+++	+
20 (67/M)	+++	++
21 (60/M)	+++	+
22 (61/F)	++	+
23 (55/F)	+++	+
24 (45/M)	+	+
25 (62/M)	++	-

The GFAP and NSE staining was performed in the fixed glioma specimens from the same surgical procedures as those used for generating viable cells. The staining was semi-quantitatively evaluated. +++: markedly positive staining; ++: moderately positive staining; +: low positive; −: no positive staining in the tumor.

**Figure 3:**
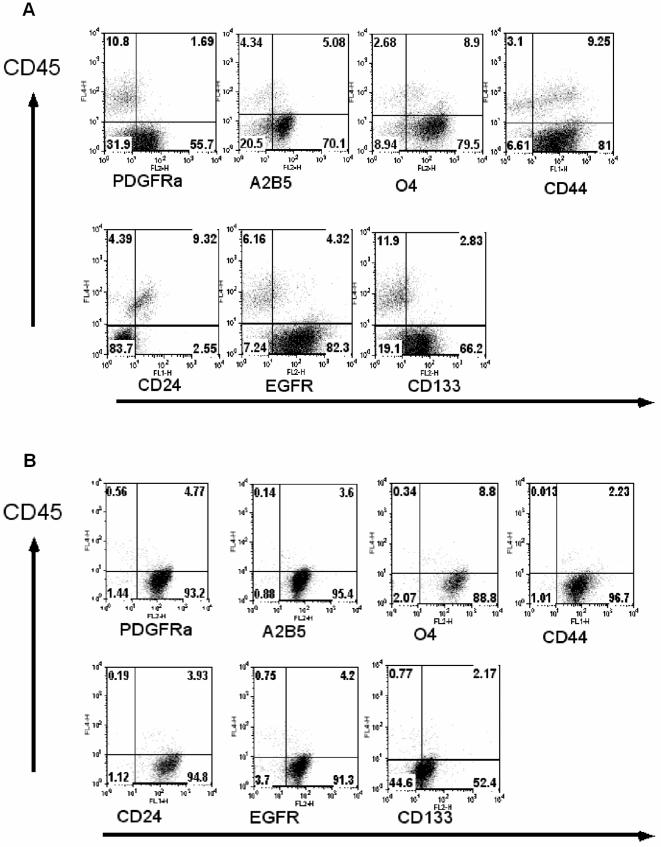
Maintenance of glial progenitor-like phenotype, but enhanced CD133 expression in high-grade gliomas. In addition to multiple glial progenitor cell markers, a high proportion of glioma cells co-expressing CD133 was detected in most of the high-grade glioma specimens. Dot-plot profiles of glial progenitor cell surface markers and CD133 expression on high-grade glioma cells from representative patients (#14 (A) and #22 (B)) are shown. Freshly isolated glioma cells were simultaneously stained with the indicated antibodies. The hematopoietic cells were distinguished with anti-CD45 staining. The numbers in each quadrate represent the percentages of the cells stained positively or negatively by the respective antibodies.

As in low-grade glioma cells, high-grade glioma cells analyzed in this study maintained the expression profiles for PDGFRα, A2B5, O4 and CD44 ( Table 1 and Figure 3). In all these high-grade glioma cases, GFAP expression was detected with inter- as well as intra-glioma variation (Table 2 and Figure 2). The expression of CD24 was observed in 7 of 16 high-grade gliomas analyzed. In striking contrast to the low-grade glioma cells, distinct cell populations with high intensity of CD133 expression (ranging from 22 to 90% of the CD45^−^ cell fraction) was detected in 12 of the 17 high-grade glioma specimens. In addition, EGFR over-expression was detected in 11 of the 17 high-grade glioma specimens analyzed. Of note, no detectable difference in cell surface marker expression profile was observed between the secondary (patients ID 15 and 16) and *de novo* GBM specimens analyzed (Table 1).

**Figure 4:**
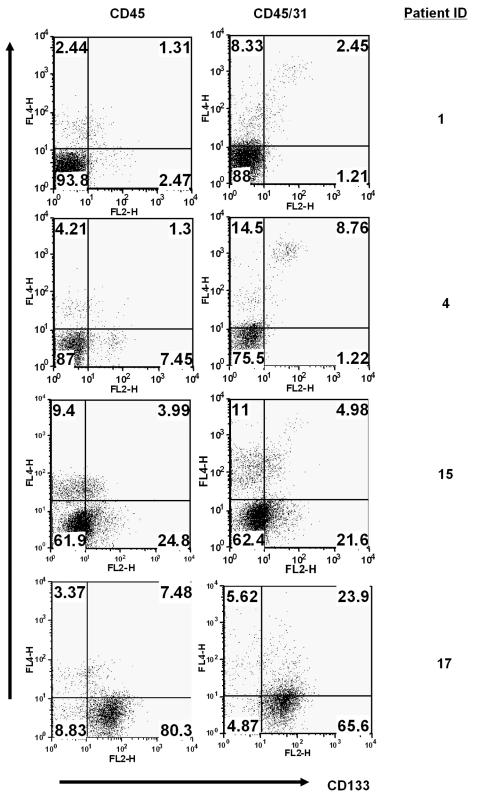
Vessel or glioma origin of CD133 expressing cells. In contrast to high-grade glioma specimens, CD133 expressing cells detected in low-grade glioma specimens are predominantly derived from blood vessel endothelial cells. Dot-plot profiles of CD133 expression versus CD45 and/or CD31 expression of cells from glioma specimens of indicated patients are shown. The numbers in each quadrate represent the percentages of the cells stained positively or negatively by the respective antibodies.

As gliomas, particularly the high-grade gliomas, are highly vascularized, newly formed blood vessel endothelial cells may also express CD133 [39]. To ascertain the glioma origin of the CD133^+^ cell fraction in our low-grade and high-grade glioma specimens, we subsequently performed CD133 expression analysis in combination with CD45 and CD31 staining. By doing so, it is possible to distinguish glioma cells from both hematopoietic cells (via CD45 expression) and tumor blood vessel endothelial cells (via CD31 expression) [40]. As shown in Figure 4, a small population of CD45^−^, but CD133^+^ cells can be detected in low-grade glioma specimens. Relatively higher frequencies of such cells were found in GBM specimens. However, such CD45^−^CD133^+^ cells were predominantly CD31^+^ in low-grade glioma specimens (ranging from 0.3 to 7% of the total living cells, n = 4). This indicates that CD133^+^ cells, if present in low-grade gliomas, are predominantly derived from newly formed blood vessel endothelial cells, and not from the glioma cells. In GBM specimens, the majority of CD133^+^ cells were of glioma origin, although CD31^+^CD133^+^ cells (ranging from 0.5 to 10% of the total living cells, n = 9) were also found.

**Figure 5:**
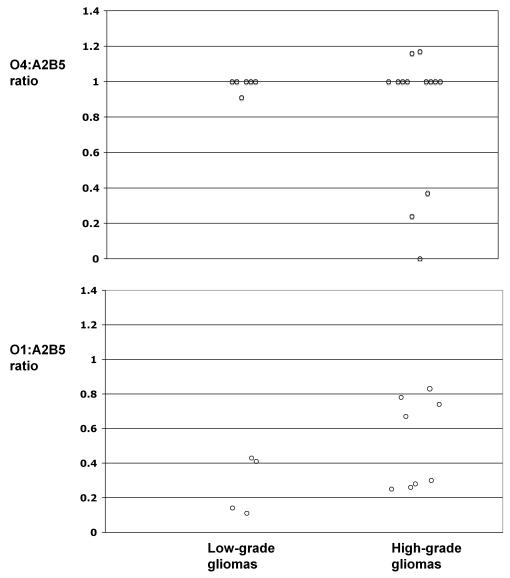
Glioma cells are compromised in downstream differentiation at the O4 stage. Glioma cells were stained with APC conjugated anti-CD45 and anti-CD31 mAbs in combination with staining for A2B5, or O4 or O1. Data shown are the ratio of the percentages between O4^+^/CD45^−^CD31^−^ and A2B5^+^/CD45^−^CD31^−^ phenotype or between O1^+^/CD45^−^CD31^−^ and A2B5^+^/CD45^−^CD31^−^ phenotype in each patient. The ependymoma patient was not included in this analysis.

In agreement with previous studies demonstrating glioma expression of oligodendrocyte progenitor cell transcription factor Olig1/2 and surface antigen NG2 [1]–[3], the concomitant expression of A2B5 (a marker for multiple types of progenitor cells in glial lineage [20]–[27], [41]), PDGFRα (a marker for early oligodendrocyte progenitor cells at O-2A stage [22], [25], [26], [42]–[44]) and O4 (a marker for late oligodendrocyte progenitor cells [29]) suggests that glioma cells, irrespective of their morphological characteristics, are committed to the oligodendrocyte lineage. To further characterize the differentiation stages along the oligodendrocyte lineage downstream of the A2B5 expressing glial progenitor cell level, we analyzed the expression of O1, a marker for pre-myelinating oligodendrocytes [29]. In contrast to the high level A2B5 and O4 expression, the frequency of O1^+^ cells were significantly diminished in all low-grade and the majority of GBM specimens analyzed (Table 1 and Figure 5). The expression of GFAP and these oligodendrocyte progenitor surface markers suggests that along the oligodendrocyte lineage differentiation hierarchy, A2B5^+^/PDGFRα^+^ glioma cells were compromised in differentiation downstream of A2B5/O4, but upstream of the O1 stage.

To study glioma cell differentiation potential along neuronal lineage, we performed an immunohistochemical staining for pan-neuronal marker NSE and synaptophysin expression in archival specimens (Table 2 and Figure 2). In low-grade gliomas, no obvious expression of NSE and synaptophysin was detected, but the cells homogeneously expressed GFAP (Table 2 and Figure 2). We were unable to ascertain whether the few cells weakly stained for NSE were the intermingled normal neuronal cells or the glioma cells (Figure 2, upper panel). In high-grade glioma cells, a regional NSE expression was clear in all cases analyzed (Table 2 and Figure 2), although both region-dependent and homogeneous expression patterns were detected. Synaptophysin positivity was detected in 7 high-grade gliomas. These data, in combination with the expression of GFAP and multiple oligodendrocyte lineage progenitor surface markers (Tables 1 and 2, Figures 1, 2 and 3), suggest that compared to low-grade glioma cells, high-grade glioma cells maintain glial progenitor-like features, but additionally exhibit enhanced CD133 expression as well as neuronal differentiation potential.

Transformed glial progenitor-like cells can provide a niche environment recruiting normal stem cells or progenitor cells into the tumor mass [45]. To investigate whether freshly isolated glioma cells studied here indeed contained transformed cells, karyotype analysis was performed in short-term cultured glioma cells (Table 3). We only observed normal karyotype for cells derived from patients 3 and 8. Abnormal karyotypes were observed from cells derived from patients 4, 12, 13, 14, 17, 18 and 20. Of note, cultures derived from low-grade glioma patient 4 and high-grade glioma patient 12 contained about 40% of the cells with a normal karyotype, contrasting the nearly homogenous phenotype between all cells (Figures 1, 3); but cultures derived from high-grade glioma patients 13, 14, 17, 18 and 20 predominantly contained cells with abnormal karyotypes. In addition, fresh cells from patients 14 and 20 formed xenograft glioma following subcutaneous injection into SCID mice [46]. Thus, the analyzed glioma cells did contain neoplastic cells and the karyotype normal and abnormal glioma cells showed hitherto indistinguishable phenotype.

**Table 3:** Karyotype analysis of short-term cultured glioma cells

Patient ID	Karyotype
**Low-grade glioma**	
3	normal
4	45,X,-Y[13]/46,XY[10]
**high-grade glioma**	
8	normal
12	45,X,-Y[11]/46,X,-Y,+7[3]/46,XY[10]
13	41-42,X,-Y,der(1)t(1;17)(p36;q21),add(4)(q31),
	del(6)(q21),+7,-10,-11,-13, 14,?dup(14)(q12q22),-15,
	-17,+2mar,inc[cp9]/80-84,idemx2[cp16]
14	44-45,X,-X,+ider(7)del(7)(q35),-10,-13,del(14)(q22),
	del(16)(q21)[cp9]/43,X,-X,der(1)t(1;14)(p34;q22),-4,
	der(6)t(6;17)(q23;p11),+ider(7)del(7)(q35),der(8)t(8;9)
	(p21;?), idic(9)(?p11;q22),-10,-13, del(16)(q21),
	der(17)t(6;17)(?;q11),
	der(17)t(13;17)(q12;p13)[cp2
17	45,X,-Y[14]/46,X,-Y,+7[3]/46,XY[6]
18	44,XX,t(9;16)(p23;q12),del(10)(p12),der(12)
	t(12;14)(p13;q12),-13,-14,dmin[16]/46,XX[9].
20	90,XX,-Y,-Y,-1,-1,+7,-8,-8,der(14)der(14)t(1;14)(q12;q22)
	x2,1-30dmin[cp 3]/45,X,-Y[11]/46,XY[18]

Freshly isolated glioma cells were cultured and analyzed for karyotype between passage 3 to 4. The number in brackets represents the number of the indicated karyotype.

**Figure 6:**
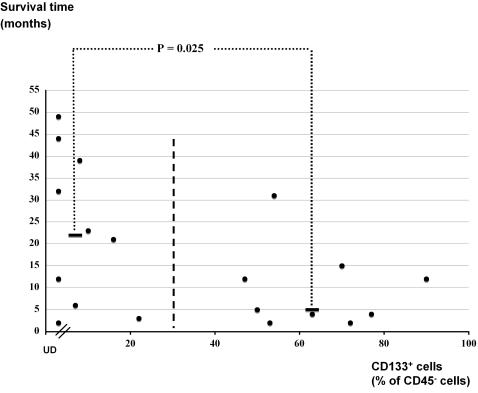
CD133 expression correlates inversely with grade II to IV glioma patient survival time. The survival time calculated from the day of operation was plotted against the percentage of CD133^+^ cells in the CD45^−^ cell fraction from the specimens of each patient. UD: undetectable CD133 expression. Bold black bars indicate the median survival time for patients in groups with CD133^+^ cells either lower or higher than 30% of total CD45^−^ cells.

Finally, we investigated whether the glial progenitor or stem cell markers expressed on glioma cells could serve as prognostic markers for patient survival. Glioblastoma patients receiving immune therapy and patients with pilocytic astrocytoma or ependymoma, were excluded from the prognosis analysis. We divided all analyzed grade II to IV glioma patients into a CD133^+^ low group (CD133^+^ cells less than 30%) and a CD133^+^ high group (CD133^+^ cells higher than 30%). The median survival time in the CD133^+^ high group was 5.0±9.2 months (n = 9) compared to more than 22.0±17.3 months (n = 10) in the CD133^+^ low group (*P* = 0.026, *t* test; Figure 6). Thus, CD133 expression inversely correlates with glioma patient survival time.

## Discussion

In this study, we investigated the expression of multiple cell surface markers characteristic of glial genesis hierarchy in freshly isolated viable glioma cells as well as the expression of GFAP, NSE and synaptophysin in the fixed specimens from a cohort of low-grade and high-grade glioma patients. Our data show that nearly all low-grade glioma cells from the analyzed cases concomitantly express multiple cell surface markers for glial progenitor cells, such as PDGFRα, A2B5, O4, and CD44. The expression of these glial progenitor markers is maintained in the high-grade gliomas analyzed. GFAP expression was detected, as anticipated, in all low- and high-grade gliomas analyzed, but the expression of NSE and synaptophysin was only detected in high-grade gliomas. Co-staining of CD31 suggests that the rare CD133 expressing cells in low-grade gliomas are predominantly of vessel endothelial origin. In more than 50% of the high-grade glioma cases, distinct CD133 expression was detected in cells concomitantly expressing multiple glial progenitor markers. Thus, low-grade glioma cells may resemble the expansion of A2B5^+^ glial progenitor-like cells; high-grade gliomas, in addition to the expansion of A2B5^+^ glial progenitor-like cells, may also contain CD133^+^ putative glioma stem cells that concomitantly express multiple surface markers of glial progenitor cells. Irrespective of CD133 expression, high-grade glioma cells are endowed with multi-lineage differentiation potential *in vivo*. Importantly, CD133 expression negatively correlated with the glioma patient survival time.

Conserved mechanisms across organ development/homeostasis and tumorigenesis have been demonstrated in many types of human cancers [47]. It is likely that gliomas may originate from the primitive cells along the postnatal glial genesis hierarchy. In fact, glial progenitor cells were demonstrated to be more susceptible to oncogene transformation compared to mature astrocytes [48]. Glial genesis originates from neural stem cells via multiple types of intermediate glial precursors [49]. In adult human CNS, putative neural stem cells have been identified in restricted brain regions [50]. In contrast, A2B5 and PDGFRα expressing O-2A progenitors are relatively abundant throughout the CNS [24], [25], [41], [51]–[53]. The aforementioned A2B5^+^O4^−^PDGFRα^−^ GRPs and A2B5^+^O4^−^PDGFRα^−^CD44^+^ ARPs may also exist. In addition to well-established PDGFRα expression, we identified that the majority of low-grade astrocytomas and high-grade gliomas concomitantly express multiple markers of glial progenitor cells such as A2B5 and O4. Our findings seem not consistent with previous reports that astrocytomas could be divided into A2B5^+^ and A2B5^−^ lineages [54], [55]. This discrepancy is likely caused by the fact that the eiptopes recognized by the A2B5, O4 and O1 antibodies are not well maintained in formalin fixed, paraffin embedded specimens, and our studies were performed in living glioma cells. It is unlikely that the co-expression of these cell surface markers is a consequence of a cell type dependent/independent program inherent to all glioma cells, rather it reflects the differentiation stage(s) common to normal glial genesis hierarchy and glioma cells. In line with previous reports identifying oligodendroglial lineage markers such as Olig2 or NG2/PDGFRα as universal markers in diffuse gliomas [1]–[3], our study demonstrates the shared pattern of concomitant expression of multiple surface markers between glioma cells and oligodendrocyte progenitor cells, which suggests that most of the morphologically diagnosed astrocytoma and GBM are endowed with oligodendrocyte lineage differentiation potential. The data of weak O1 expression, but homogenous GFAP expression, indicate that these cells are likely blocked at differentiation pathways between O4 and O1 stages in oligodendrocyte lineage or at hitherto unidentified stages towards type-2 astrocytes [20], [21], [28].

The expression of CD44 identifies the ARPs [28] and nearly all gliomas express this marker. This CD44 expression does not necessarily contradict the oligodendrocyte lineage commitment of A2B5^+^PDGFRα^+^O4^+^ glioma cells which exhibited a concomitant GFAP expression, because a misexpression of CD44 can result in expansion of oligodendrocyte progenitor cells with impaired maturation and concomitant gain of GFAP expression [28]. However, it cannot be excluded that A2B5^+^CD44^+^GFAP^+^ glioma cells without detectable PDGFRα and O4 expression (e.g.: case #9, Table 1) actually represent the expansion of ARP-like cells.

Previous neurosphere culture-based studies have demonstrated that sphere-forming glioma cells are capable of multi-lineage differentiation [8], [9], [56], [57]. A potential inherent weakness in this type of studies, however, is that neurosphere culture studies only assess the particular fractions of glioma cells, which are supported by the neurosphere culture conditions [10], and the neurosphere culture conditions do not necessarily represent the glioma niche environment. Additionally, multi-lineage differentiation capacity can also reflect the differentiation plasticity induced by neurosphere culture conditions. It has been demonstrated that lineage-restricted progenitors gain multi-lineage differentiation capacity in neurosphere-forming assay [11], [58]. We demonstrate that high-grade glioma cells, in contrast to low-grade glioma cells, can express neuronal marker as well as CD133 *in vivo*. Thus, our data suggest that the *in vivo* multi-lineage differentiation capacity is likely restricted to high-grade glioma cells. It is unclear whether the *in vivo* multi-lineage differentiation capacity can be gained following the progression from low-grade to high-grade gliomas.

Gliomas are composed of a mixture of neoplastic and non-neoplastic cells [45]. The non-neoplastic cells could include entrapped neurons, astrocytes, microglial cells, blood vessel cells. It is also demonstrated in mouse glioma models that normal progenitor or stem cells can be recruited to glioma niche [45]. Although the possibility could not be entirely excluded that the cells with normal karyotype or isolated numerical aberrations are also neoplastic, our data suggest that most of the entrapped cells in human gliomas show a progenitor or stem cell-like phenotype and such cells can constitute more than 40% of the total cells in low-grade gliomas, but their frequency varies substantially in GBM specimens. It remains to be established whether neoplastic and non-neoplastic cells in gliomas can be phenotypically distinguished.

Considering gliomas as a group of progressive tumors, our data demonstrate that expression of the CD133 associated stem cell features is correlated with a poor prognosis. High-grade glioma patients without detectable frequencies of CD133 expressing cells as well as grade II glioma patients appeared to survive significantly longer compared to high-grade glioma patients with high frequencies of CD133 expressing glioma cells. This finding extends previous studies that “stem cell-ness” gene expression pattern can serve as a marker for poor prognosis of malignant diseases [59], [60]. Our findings are consistent with a recent report on a correlation between enhanced CD133 expression and a poor clinical outcome in glioma patients [61]. Importantly, we show that the rare CD133 expressing cells in low-grade gliomas are predominantly of blood vessel origin. Of note, the frequencies of CD133 expressing cells in high-grade gliomas analyzed in our studies appeared to be much higher compared to previous reports [7], [56], [61]. This discrepancy could be because we analyzed the CD133 expressing cells in CD45-negative cell fraction and glioma specimens can have different contents of hematopoietic cells.

This study constitutes an important step towards clarifying the lineage commitment and differentiation blockage of glioma cells. We have demonstrated that normal glial progenitor cell surface markers are widely expressed in glioma cells. Glial progenitor cell surface markers could potentially be used to design glial progenitor antigen-targeted immune therapy or gene therapy. As normal neural stem cells are believed to be devoid of glial progenitor cell surface markers, targeted delivery of cytotoxic agents to selectively ablate glioma cells would spare the normal neural stem cells.
